# Preventing and countering insider threats and radicalism in an Indonesian research reactor: Development of a human reliability program (HRP)

**DOI:** 10.1016/j.heliyon.2023.e15685

**Published:** 2023-05-03

**Authors:** Djarot Sulistio Wisnubroto, Khairul Khairul, Fatmuanis Basuki, Endang Kristuti

**Affiliations:** National Research and Innovation Agency of Indonesia – BRIN, Puspiptek, Serpong, Tangerang Selatan, Indonesia

**Keywords:** Human reliability, Insider threat, Radicalism, Security, Safety, Research reactor

## Abstract

Nuclear technology has been present in Indonesia for more than 60 years, with the main facilities being three research reactors managed safely and securely. Considering the rapid changes in Indonesia's socio-political and economic situation, it is imperative to anticipate potential insider threats due to these influences. Thus, the National Nuclear Energy Agency of Indonesia developed the first human reliability program (HRP) in Indonesia, perhaps the first HRP in Southeast Asia. The development of this HRP was based on qualitative and quantitative analysis. The HRP candidates were determined based on their level of risk and ability to access nuclear facilities, and 20 people who worked directly in a research reactor were selected as HRP candidates. The candidates' background data and interviews were the basis for determining their assessment. The 20 HRP candidates were unlikely to be an internal threat. However, some of the candidates had significant records of job dissatisfaction. Counseling support could be one of the solutions for this issue. Two candidates disagreed with government policies, so they tended to sympathize with banned groups. Therefore, management should warn and nurture them to not become future insider threats. The results of this HRP provided an overview of the HR situation in a research reactor in Indonesia. Various aspects must be further developed, especially management's commitment to periodically or when necessary, increase the knowledge of the HRP team and, if necessary, invite outside experts.

## 1 Introduction

Human reliability is often used in fields that require high safety standards, such as chemistry, petroleum, aviation, and nuclear utilization activities. Complex human behavior always has the potential for causing inherent risk against the background of various triggers that can cause errors in operating systems or processes. Therefore, the human factor can both positively or negatively impact performance at work. Generally, human error can be minimized through education and training/retraining programs, however, some worker (insider) actions can be intentional, endangering safety and security in the workplace due to personal, economic, ideological, or political motivations. The Human Reliability Program (HRP) is expected to ensure that individuals that occupy positions with access to critical facilities, activities, or locations, meet the highest standards. To ensure that they comply with work rules and safety and security regulations to be reliable workers, trustworthy individuals, as well as physically and mentally stable.

For more than 60 years, the National Nuclear Energy Agency of Indonesia (BATAN) has managed many nuclear facilities, including the Bandung research reactor, which has been operational for 57 years, a 52-year-old reactor in Yogyakarta, and a 34-year-old reactor in Serpong. BATAN also has various other facilities, such as radioactive waste management facilities, nuclear fuel fabrication facilities, and radioisotope-radiopharmaceuticals production in safe and secure conditions. In addition, despite budget constraints as well as aging and decreasing human resources, the performance of all nuclear facilities has been satisfactory. To date, there has never been a radiation or nuclear accident that can endanger humans, public, and environment. However, on September 10, 2007, there was a chemical explosion in one of the laboratories in Serpong. A group of researchers carried out biofuel experiments that had nothing to do with radioactive or nuclear material. The accident is a critical lesson on improving the safety culture among employees. Then, in 2012, a program to improve safety culture was initiated by conducting a safety culture self-assessment. This annual self-assessment had a significant impact on the safety system in BATAN [1,2]. Almost simultaneously, the activists of nuclear security culture also began a similar self-assessment. In order to equalize the existence of safety and security, BATAN in 2012 carried out a security culture self-assessment. So that security culture activities are institutionalized, then in 2014, the Center for Security Culture Assessment (CSCA) was established in collaboration with Georgia University in the United States of America [3]. The biggest challenge in developing these safety and security cultures in BATAN is aging human resources. The average age of BATAN's employees is more than 47 years, so most of them are in their comfort zone. This is consistent with several studies on civil servants in Indonesia, which suggest that the longer they work in a particular position, the more difficult it becomes for them to leave their comfort zone [[4], [5], [6]] [[4], [5], [6]] [[4], [5], [6]]. Consequently, any type of change would require time, motivation, and consistent enforcement of the rules.

On the other hand, in the last ten years, there have been increasing concerns about extremism and radicalism amongst the public and Government employees [7] including in BATAN, as this agency manages high-risk facilities such as nuclear reactors and many radioactive sources categoriy I and category II. Although so far, from general evaluations, there has been nothing suspicious at BATAN. Nevertheless, all aspects that have the potential to be insider threats must be immediately known, for example, deviant beliefs/ideologies, dissatisfaction at work, family and environmental problems, and effects from health conditions.

Indonesia has experienced several terrorist attacks over the last twenty years, including the terrorist bombings at tourist sites in Bali in 2002 and 2005, which killed hundreds of people [8,9]. Even during the pandemic in March 2021, there was a terrorist attack at the police headquarters in Jakarta [10]. To prevent further acts of terror, the Indonesian Government focuses on implementing deradicalization programs for people exposed to radicalism, education and outreach through social media and mass media, and establishing a terrorism prevention coordination forum involving communities throughout Indonesia [11]. However, the Indonesian Government does not yet have a specific program to prevent the dangers of extremism and radicalism amongst internal government employees (civil servants/ASN or *Aparatur Sipil Negara*) [12], the deradicalization program has been carried out by the government for targeting non-state actors only. Although recently, the Indonesian Government has issued a ban on civil servants issuing hate speech (including on social media) regarding sensitive issues such as religion, race, and intergroups [13]. Nevertheless, there is no specific program to prevent the potential threat of extremism, nor is there a program to prevent insider threats in strategic facilities.

Therefore, the National Nuclear Energy Agency of Indonesia – BATAN (since 2021, BATAN has been integrated into a new organization called the National Research and Innovation Agency (BRIN or *Badan Riset dan Inovasi Nasional*), as the organization that manages nuclear facilities in Indonesia, initiated the development of the Human Reliability Program (HRP). Among its objectives, apart from ensuring the safety and security of nuclear facilities, it could also form an example for other strategic facilities in Indonesia and other developing countries to prevent insider threats and, more broadly, prevent the dangers of extremism and radicalism.

In addition, like many nuclear facilities in various countries [14], BATAN was experiencing the aging of its employees, which can have implications for possible demotivation related to increased risks to the safety and security of the nuclear facilities it manages. This HRP activity can be one of the evaluation keys to improving human resource management in the future. This study provides an overview of the stages of HRP implementation in Indonesia for the first time. Challenges faced and possible development in the future.

## 2 Literature review

Human Reliability Programs (HRP) for nuclear facilities originated in the USA in the 2009 era because they managed around 104 nuclear power plants and needed to anticipate insider threats. At the 2016 IAEA International Conference on Nuclear Security: Commitments and Actions, the United States issued IAEA Information Circular, INFCIRC/908, “Joint Statement on Mitigating Insider Threats,”. This step is a joint effort to prevent insider threats at nuclear facilities and other related facilities. The country is experienced in implementing HRPs, especially in sensitive facilities. The HRP aims to ensure that individuals who work in a sensitive facility (such as a reactor, handles radioactive waste, nuclear fuel, and explosive materials) are physically and mentally capable to work in such conditions. The HRP emphasizes competency issues as well as physical and spiritual health conditions, including a history of alcohol use, drugs, legal and illegal drugs, and other things that might disrupt workers' performance in terms of safety and security [15,16]. The HRP is imposed on personnel who are considered to have the potential to be quite vulnerable to misusing their authority, for example, operators and supervisors of the reactors. Even high-level directors can potentially be vulnerable, therefore the HRP must be periodically applied throughout every level of the organization. The HRP itself includes reviews by superiors, psychological evaluations, credit checks/financial review, evaluation of medical check-up results, and a review of performance from a security aspect. The implementation of the program must be as transparent as possible, it should use written methods and interviews, and be preceded by explaining the program's benefits.

Insider threats are posed by individuals within an organization or a particular security environment. In the context of nuclear reactors, insider threats can refer to threats posed by employees or contractors who have access to the facility, including access to control systems and radioactive materials. Examples of insider threats in nuclear reactors include information leaks, manipulation of control systems, sabotage, theft of radioactive materials, and the improper use of radioactive materials. Insider threats can be particularly dangerous because insiders possess knowledge and access to systems that can cause significant harm. A combination of technical and non-technical measures is required to address the insider threat in nuclear reactors. Technical measures include securing control systems and radioactive materials, monitoring employee and contractor activities, and improving physical and cyber security. Non-technical measures include training employees and contractors on insider threats, conducting rigorous background checks, and fostering a strong security culture [[17], [18], [19], [20]] [[17], [18], [19], [20]] [[17], [18], [19], [20]].

This program is estimated to effectively prevent and counteract potential actions that could seed internal threats. In Indonesia, the problem can be more complex as it could involve economic factors (e.g., salaries are too low) [21], health factors (although because most of the Indonesian population are Muslims, hence alcohol abuse is minimal) [22], and the influence of beliefs/ideology [23]. Moreover, if a person's beliefs/ideologies are suppressed, curbed, or prohibited, a form of venting could be in the form of disrupting the institution's performance [23]. Therefore, ideological factors, loyalty to laws, regulations, and policies from the Government are included in a parameter of the psychological review, both in writing and in interviews. This program is also helpful for human resources development, so the manager of a facility can consider improving its employees both in terms of technical ability, loyalty, emotion, and external influence. The final goal of the HRP program is to have employees who are reliable, honest, trustworthy, as well as physically and mentally stable so that nuclear facilities can be operated safely, securely, and reliably under applicable requirements. As an exciting reference, like Indonesia, which does not yet have a nuclear power plant, Nigeria has also been trying to develop HRP in its nuclear industry. The challenge they faced was adopting the HRP that originated in the United States into the local culture [24].

BATAN developed and piloted the HRP for the first time in Indonesia and possibly the first time in Southeast Asia. The development of this HRP is expected to provide a new perspective in dealing with potential safety and security disturbances of high-risk facilities.

## 3 Methodology

This activity was BATAN's first attempt to develop a human reliability program based on several guidelines from the Partnership for Nuclear Security (PNS) [25,26], IAEA [17,[27], [28], [29]], and Indonesian regulations [[30], [31], [32]]. This program was implemented in 2018 in a research reactor. BATAN has formed an HRP implementing team consisting of managers, nuclear safety experts, nuclear security experts, psychologists, and several medical personnel to conduct these activities. The HRP team adapted the program to the country's situation and included national insight as an indicator to counteract the issue of radicalism and extremism. This is done in parallel to conducting qualitative and quantitative studies through background analysis and interviews with research reactor employees.

This study used a deductive approach using the theory built into compiling the HRP program and a latent approach by deepening data related to the social context. This research focused on the indicators set in the HRP program to measure how reliably a person works in a high-risk facility. So even though qualitative research was carried out, it cannot fully follow the thematic analysis steps developed by Braun and Clarke [33]. In the early stages, familiarization was carried out to get to critical issues, but afterward, it focused more on the indicators set in the HRP program.

**Fig. 1:**
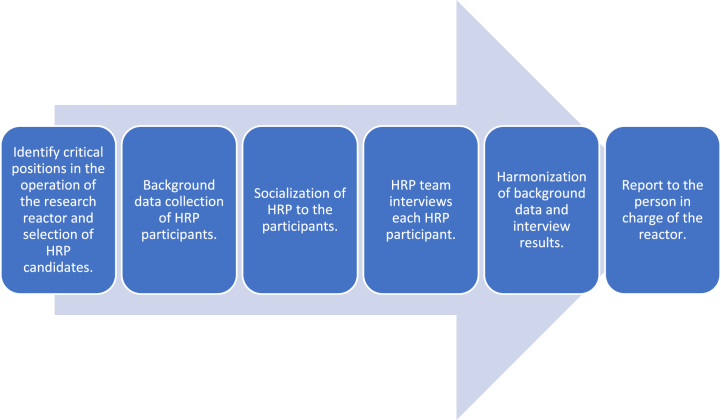
Flowchart of human reliability program (HRP).

Initially, to determine the HRP candidates, the team analyzed the characteristics of the research reactor [34], the level of risk, and the employees' level of access to the facility. The program was carried out transparently, starting with an HRP workshop by BATAN management for all participants and team members. The workshop was needed to ensure that all parties involved had the same perceptions about the program. Then, the team's first task was to evaluate the existing database of employee records. This included education and employment data, employee performance appraisals, employee salaries, history of medical examination results, drug-free examinations, family history lists, and other information from various sources that can affect an employee's performance. Each participant filled out a form to answer questions related to 10 indicators: loyalty, ideology, nuclear security, health (including drug and alcohol use), financial responsibility, honest behavior, social interaction, emotional/social stability, weaknesses, and strengths. After the participants filled out the forms, the team conducted in-depth interviews with each participant. Then, the team evaluated the reliability status of each candidate by considering the safety and security factors of the facility as well as the health, psychological, and economic status of each worker. The team then submitted a report to BATAN management to decide the eligibility of each worker to operate the research reactor. Fig. 1 shows a summary of the steps of the HRP program.

### 3.1 Selection of HRP candidates

Ethical approval for this study was obtained from the National Nuclear Energy Agency Ethics Committee – BATAN (EC approval number: BATAN/156/2018). Informed consent was obtained from all candidates before they participated in this study. Participants were informed of the research objectives, procedures, and their right to withdraw without penalty.

The HRP position is a critical personnel position that has the potential to cause severe damage to facilities and or national security as well as it has access to sensitive nuclear security information. Therefore, HRP positions need to be identified and updated whenever there is a change in position or personnel. In terms of the identification of HRP participants at research reactor facilities, supervisory managers can conduct an initial assessment and identification of attitudes and behavior of HRP participants based on the results of their annual review of personnel or based on the consideration of other personnel who have indicated their peers' decrease in reliability in attitudes and behavior towards the organization. The following factors inside and outside the reactor can help measure how critical each person's position is in operating a research reactor.

a. Reactor characteristics.
b. The physical form of the reactor.
c. Parts that pose a safety and security risk inside the reactor.
d. Nuclear material transfer system inside the reactor.
e. Reactor worker safety and security risks.
f. The risk of threats from inside the reactor.
g. Reactor facility operating system.
h. Consideration of external influences on reactor safety and security.
i. Political considerations in influencing reactor workers.
j. Process permission to enter the facility inside the reactor.
k. Direct or indirect access to the reactor (including the internet network).
l. The possibilities of sabotage of nuclear materials and facilities in the reactor.
m. Other risks from outside the reactor.

Furthermore, the candidates were selected based on their access and risk positions in the reactor facility. The definition of “*access*” is the ability to access locations, facilities, and information to carry out their activities. Access includes physical entry to a nuclear facility, nuclear materials, related systems, components, equipment, and computer systems. Access also includes remote monitoring and operating computers at a facility, such as access to computer systems and networks that control processes, provide safety, contain sensitive information, or contribute to nuclear security. Positions that have greater access to facilities and information are given a high ranking.

The definition of “*risk*” in this study is the potential undesired outcome resulting from a nuclear safety and security event as determined by its probability and the associated impact if it occurs, including consequences for people, facilities, and the environment. Risk is generally a function of three components: threat, vulnerability, and consequence.

Risk is closely related to authority, and the higher the authority, the higher the risk. The definition of “*authority*” is the power to perform operations as part of their work tasks and the power to direct other employees. Individuals with authority can support malicious actions, including physical or computer-based actions.

The ranking of the access and risk positions of the research reactor employees are shown in Table 1.

**Table 1:**
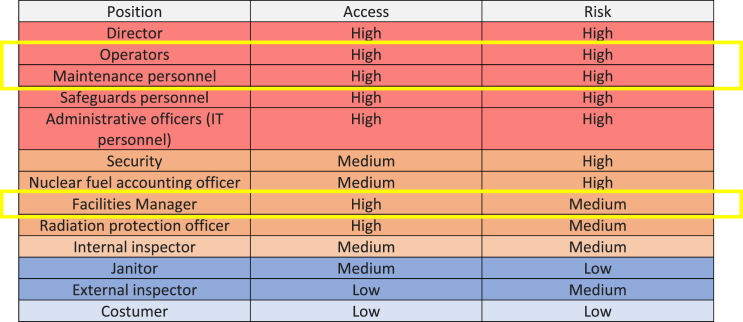
The ranking of the risk positions of HRP participants in the reactor facility.

Since this was the first HRP program implemented in a nuclear facility in Indonesia, only facility managers (including supervisors), operators, and maintenance personnel were selected as candidates in the initial HRP phase. They were chosen because they directly operated and maintained the reactor facilities. Thus, 20 personnel were the HRP candidates among 187 employees that worked at that reactor. With time, all positions, especially those with high risk, will be part of the HRP process in the next agenda.

The HRP candidates had the following obligations:

- Fill out the HRP document by signing the statement and consent to release privacy rights on the HRP document.
- Submit matters relating to mental or physical conditions or disorders that require treatment or care to the HRP Team Leader.
- Participate in HRP training
- Conduct random drug and alcohol tests.
- Provide complete and correct answers to relevant HRP-related questions to enable the HRP Team to make decisions regarding HRP certification.

Therefore, after the selection of HRP candidates, it was vital to carry out the workshop/socialization of the HRP program for all candidates and the implementing team to avoid any misunderstandings about this program. The focus of the HRP program is to avoid possible internal threats that pose a risk to the safety and security of reactor operations. Nevertheless, external factors that can influence the threats, including economic, social, political, and ideological factors were inevitably discussed. However, these external factors were discussed openly and transparently.

### 3.2 The initial evaluation

The initial evaluation of HRP candidates includes the following steps [16]:

• Examination of background conditions An examination of a personnel's conditions consists of gathering information and evaluating them through a personal interview and observing their activities on social media. Interviews were conducted to obtain information about a person's character, personality, and lifestyle.
• Drug TestDrug testing was conducted as drug addiction can affect a person's performance and would have implications for the safety and security of the facility. If an HRP candidate tests positive for drugs, they would automatically be removed from a critical position in a nuclear facility. In addition, although most Indonesian people are Muslim, random alcohol tests should be done as alcohol addiction affects a person's performance.
• Criminal record examination The track record of criminality was conducted in collaboration with the police. If a worker has ever committed theft, embezzlement, or forgery, they would be removed from work.
• Debit and Credit Check It is necessary to track the debt habits or credit use of an HRP candidate. Debt that exceeds one's ability and excessive credit card usage habits can cause a person to be unable to pay their debts or credit on time. The following result is that the person has the potential to experience extortion. This situation cannot be tolerated and is a factor for a personnel's disqualification from work.
• Education Verification Examination of education and training qualifications is critical to determine whether the person concerned has the capabilities their position requires.
• Performance Verification Examination of worker performances was conducted by checking their annual employee performance targets and yearly targets achievements. The assessment does not only cover performance targets but also includes work discipline, teamwork, and communication skills.
• Security knowledge and orientation Understanding nuclear security and its safety is an essential factor for workers. Therefore, worker evaluation of attitudes and actions in working in the field can be done, for example, by interviewing or by viewing CCTV video recordings in the workplace. Training programs can also be given to refresh and increase the worker's awareness and understanding of the facility.

During the first implementation of the HRP, the data obtained from the HRP participants' initial evaluation was generally sufficient. BATAN regularly evaluates the performance and attitude of its employees and conducts health checks for its employees every year. Nonetheless, some things need to be improved in the future. For example, there was no data on the employees' debit and credit card usage. Though, in some instances, the bank would contact the office to inquire about the employee's work status for those that are in debt. From these cases, the office obtained a record of the debt status of the employee concerned.

Besides this, Indonesia is in the top 10 active social media users globally [35] and many BATAN employees actively use social media to express their activities and opinions on events happening around them. The Government prohibits its employees from using social media to spread hate speech, write racist sentences, offend certain groups and religions, and directly or indirectly support extreme and radical groups prohibited by the government [13]. Nevertheless, the Government cannot monitor all of its employees' social media use. However, there are usually reports from the public or other employees if an employee on their social media account tends to sympathize with a prohibited radical or extreme group. Cases like this have become essential data in the initial evaluation of HRP. From the reports that came in, some candidates of HRP were suspected of sympathizing with extreme groups that the government prohibits. The HRP team then conducted in-depth interviews of the alleged employees from this information. Information on the interviews conducted is presented below.

### 3.3 The interview preparation

The focus of the interviews with HRP candidates was to explore several external factors or personal problems they face. The motivation possessed by insiders to commit crimes in the facility may come from several reasons, such as criminal, ideological, political, financial, personal, or forced and under pressure from outsiders in the form of threats, intimidation, and other security factors. Personal motivation can also arise from disappointment, frustration, dissatisfaction, resentment, and feeling neglected or unappreciated due to the following reasons:

a. Their current income does not sufficiently meet their needs.
b. A very heavy workload.
c. The type of work does not match the character of the employee.
d. There is no adequate appreciation from the management.
e. Personal or family problems that are difficult to resolve.

Disappointment, frustration, dissatisfaction, as well as feeling neglected and unappreciated will decrease the employee's performance in terms of quality and quantity. It can even affect the performance of other employees in the group. Even though in BATAN the Employee Performance Target (SKP or *Sasaran Kinerja Karyawan*) is implemented quantitatively, the overall work quality may decrease. A psychological approach by applying a human reliability program may solve this problem.

Before conducting interviews, the HRP team distributed questionnaires to its participants which focused on external and personal factors. The questionnaire consisted of several questions, and the participants must fill out a questionnaire with a minimum of 100 words. The questionnaires were then collected at the secretariat as interview material. The HRP team then cross-checked the questionnaire results with the BATAN database on the performance, salary, and health conditions of each HRP participant. At this stage, the HRP team had been able to make an overview of the performance of each HRP participant, including the possibility of strange things that could interfere with the safety and security of nuclear facility operations. For example, complaints about low salaries, feelings that there was no clear continuing education and promotion program for employees, complaints about a lack of open communication with superiors, and other sentences indicating dissatisfaction with the work environment.

So interviews were focused on exploring things that might interfere with the performance mentioned above. The interviewer explored external and internal factors not written in the questionnaire or database. Beside, BATAN has developed ten indicators for this HRP program. These indicators aim to determine whether a person has integrity, honesty, and reliability in operating a nuclear facility. Specifically, the indicator of national insight was created to anticipate the influence of radical or extreme groups that could directly or indirectly create threats from within. The following are the indicators used in the HRP:

1. Loyalty Loyal workers were hired to do a specific job and do everything they can to do their best. Dedicated workers work hard for their pay while being committed to the organization's success. Therefore, by understanding the reason behind the workers' loyalty problems, including job dissatisfaction, the organization could look for ways to overcome these issues and improve their overall performance.
2. National insight National insight means that all people or groups put the interests of the state and nation first. Asking about the workers' national insight is important as it also determines their standing on placing the unity and safety of the country above the interests of individuals or groups.
3. Understanding of nuclear security Knowledge of nuclear security is essential for workers in research reactors. Nuclear security activities are meant to prevent, detect, and act against sabotage, theft, illegal transfer, unauthorized access, or other malicious acts involving nuclear materials, other radioactive substances, or related nuclear facilities [36].
4. Health, drug, and alcohol use The organization is very concerned about the health of its workers. Understanding the signs of drug and alcohol abuse will help the organization manage health and safety risks at nuclear facilities.
5. Financial responsibility The Government has provided sufficient salaries for every person working in nuclear facilities. However, specific reasons can cause someone to experience financial difficulties that interfere with their performance and pose a safety and security risk in the workplace. Thus, this is an important indicator for the HRP.
6. Honesty Honesty is one of the main principles in team building and is a fundamental principle in maintaining the safety and security of a facility. Honest team members should be able to tell the truth about problems without any fear or worry so the team can resolve them before it escalates.
7. Obedience In principle, the benefits of rules and regulations in nuclear activities protect workers, the public, and the environment. Therefore, following rules and regulations would help all personnel understand what is expected from them and what will happen if they violate the rules. It makes for a stable workplace environment where people feel safe to come to work, be themselves, and do their activities. This result is less turnover, more teamwork, and higher morale.
8. Social interaction Personnel with social connections at work tend to be more engaged and loyal. Quality working relationships help build a solid organizational culture that emphasizes respect, loyalty, and trust. It provides a sense of cohesion essential to fostering creativity, teamwork, and collaboration.
9. Emotional stability Emotional stability is defined as an individual's ability to remain calm under various pressures. Emotionally unstable people are more explosive, which results in an increased risk of behavioral harm. A person's ability to avoid emotions and the extent to which a person experiences anxiety in certain situations is determined by emotional stability. This is related to a person's ability to cope and adapt to various life situations. Thus, this is an important indicator for the HRP.
10. Self-awareness When people see themselves clearly, they are more confident and creative. They make sounder decisions, build stronger relationships, and communicate more effectively. They are less likely to cheat, lie, and steal. They are better workers who get more promotions. Therefore, these positive attributes are one of the indicators of physical and mental health needed to work in a research reactor.

These ten indicators formed the basis of interviews with HRP candidates. As mentioned above, the interviewer would also develop a specific discussion according to the questionnaire written by the candidate and the relevant data provided by the organization.

After 2 team members interviewed each HRP candidate, the interviewer assessed according to the criteria in Table 2 below. All team members then validated the interview results by harmonizing with existing data and inviting health, security, safety, and technical experts. The validation includes analyzing and identifying records/reports of each indicator from each HRP participant. If there is a questionable conclusion regarding the candidate assessment during the validation process, the team will vote.

**Table 2:** The HRP participants’ interview score criteria.

Scoring	Criteria
91–100	Excellent: the probability of an insider threat for this personnel is very low. Personnel has excellent responsibilities (reliable, healthy, high performance, reliable).
81–90	Good: this personnel has a low probability of insider threat. Personnel has a reasonable sense of responsibility.
71–80	Fair: this personnel needs monitoring and regular evaluation. This personnel has potential tendencies to be an insider threat.
60–70	Poor: this personnel needs to be monitored and their access to certain facilities should be restricted. Such personnel should be suspected of being a potential insider threat.
<60	Very poor: this personnel is not allowed to work in the control area and is identified as an insider threat.

However, if a good average result for all participants is obtained from using the assessment criteria at the time of the interview, the team agreed to use another assessment method where the average (Mean), the highest score (HS), the lowest score (LW), and standard deviation (Std) of the 10 (ten) indicators from each participant are calculated. Next, the mean plus the standard deviation (Mean + Std) was calculated as the Upper Limit and the mean minus the standard deviation (Mean – Std) was the Lower Limit. The category assessment would be based on the following categories:

a. If the value is between the Upper Limit Value and the Lower Limit Value, then it is declared normal (green category);
b. If the value is below the Lower Limit Value, it is declared low (yellow category);
c. If the value is above the Upper Limit Value, it is declared deficient (red category).

## 4 Results and discussion

The 20 HRP candidates in this study worked on an old research reactor that has been operational for more than 30 years. The supervisors, operators, and maintenance officers were divided into three daily shifts. Six supervisors were senior employees and were over 50 years old, all of whom have extensive experience and knowledge in handling the research reactor. Meanwhile, young personnel (most of whom are under 30 years old) were in the position of operators. The 7 maintenance personnel in this study had similar characteristics to the supervisors, they were generally senior employees over 50. Of all the HRP candidates, 12 people were over 50 years old, and 5 people were under 30 years old. This gap will cause a big challenge in regenerating personnel at the reactor soon.

Overall, as shown in Table 3, all HRP candidates scored above 80. This result indicates that there is little chance of an internal threat. However, when examined more deeply, based on the interviews and data collected, there were some significant findings as the results suggest that some personnel require treatment from health, psychology, and security experts. This finding is also indicated by some of the below-average scores, but none of the scores were in the red category.

**Table 3:**
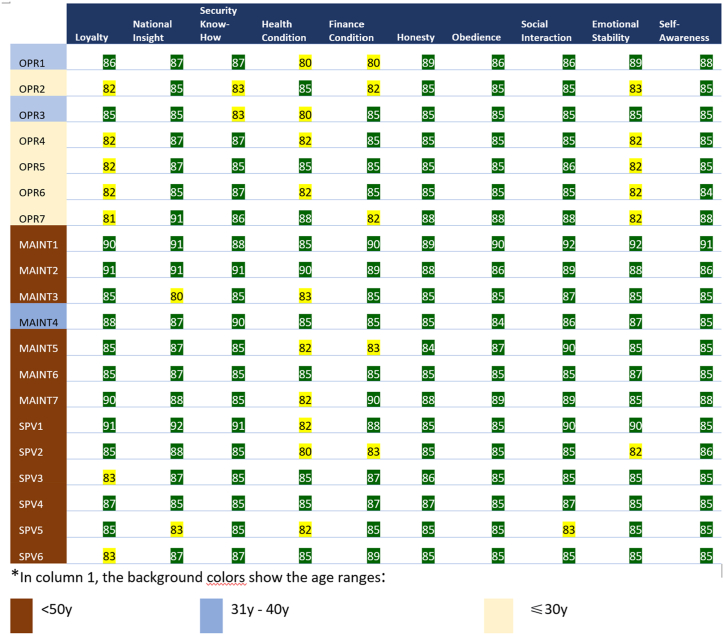
The HRP Results for Senior Employees of a Nuclear Facility.

Health condition occupies the most significant percentage of the relatively low scores, i.e., 50% of employees require health attention. The results also found that 35% of employees have a minor job dissatisfaction (loyalty) record, 30% of employees require attention to their emotional stability, and 25% of employees have financial responsibility problems. Additionally, 10% of employees require support in terms of national insight and knowledge of nuclear security.

In the loyalty indicator, the participants had an average score of 85.4 (eighty-five point four), which falls in the good category. These results illustrate that HRP participants are committed to their work and are highly loyal to the institution, as shown by their willingness to work outside office hours and seriousness about working to improve their performance. However, there is a tendency that the younger the employee's age, the lower the loyalty score. Uniquely, two supervisors also obtained scores that are below the average or are marked with yellow marks.

The yellow category on this loyalty indicator is related to job dissatisfaction expressed by 7 (seven) participants. Generally, they conveyed an attitude of disappointment, dissatisfaction, a sense of injustice, and were feeling neglected or unappreciated. Young employees wanted clarity on career paths and transparency of educational programs, including overseas training and awards for outstanding employees. Meanwhile, senior employees wanted better communication with management and comparative studies with other facilities to gain new experience and knowledge.

One of the reasons the HRP program was implemented was because of fears of radical ideas infiltrating personnel working at nuclear facilities. Several reports indicated that some employees favor banned groups on their social media. Studies that were conducted in Indonesia revealed that several key factors influence the process of individual interaction with radical groups and ideologies. The main factor is personal problems such as boredom at work, including not knowing the purpose of working or a working system that they think is contrary to their beliefs. This saturation point then encourages individuals to move towards a deeper understanding of religion which is expected to provide a meaningful life purpose and/or identity for them and reduce their existential anxiety. This situation eventually led individuals to join radical online and offline groups and, over time, increases their participation in these group activities [9].

Fortunately, regarding national insight and the possibility of radical infiltration, no employee can be considered a deviant, and nor do they have the potential to become an insider threat. The two individuals who scored relatively low in national insight are generally critical of various Government policies in the socio-economic and political fields, but not in their rebellious attitude towards institutions. This critical attitude may result from the polarization among some Indonesians after the 2014 presidential election. Since the 2014 presidential election, then the 2017 gubernatorial election in Jakarta, and finally the 2019 presidential election, the rivalry between President Joko Widodo (Jokowi), whom pluralist groups back, and his former opponent, Prabowo Subianto, whom Islamists back, has fueled the previously invisible political divisions between the two groups. There are still frictions in society between these groups, primarily via social media [37]. In terms of loyalty indicators, the participants in this study had high scores. However, continuous monitoring is still required to prevent unwanted actions. Until now, management has often opened dialogues with the two individuals with low scores about the safety and security of nuclear facilities and provided directions to avoid any dangerous opinions on government policies. Management also prohibits them from liking or being directly or indirectly involved in prohibited groups.

In terms of knowledge and awareness of nuclear security, almost all HRP participants stated something in common about what nuclear security was and what actions must be taken to prevent and overcome anomalous situations related to security. However, there was two personnel who were labeled yellow. These individuals asked the interviewer various questions about the importance of nuclear security culture and were also critical of several examples of inconsistencies in the implementation of nuclear security in the field. This input provided a positive take for management to correct these criticisms, and the two personnel were proposed to take part in a nuclear security culture training which is often held in Indonesia.

In Indonesia, government agencies rarely conduct annual medical examinations for their employees. However, BATAN received an exception. The health of nuclear facility workers was a priority for the Government. In addition to obtaining national health insurance coverage, every nuclear facility worker must conduct an annual health check-up, receive an additional salary as a form of protection from nuclear hazards, and receive a package of nutritional supplements and vitamins every month. Government employees generally do not accept these packages. However, BATAN wanted its workers to always be in good health at work, which will reduce safety and security risks. Therefore, the organization has complete data on the health condition of each worker and the health data for all employees helped facilitate HRP activities.

The result of the health data analysis of every HRP candidate suggested there was nothing to worry about in terms of the workers' health. No employee was addicted to drugs or alcohol. Although the participants were declared healthy to work, 10 workers received a note from the medical officer. Their medical records indicated that they had relatively high cholesterol levels, symptoms of diabetes, hypertension, gastritis, smoking habits, and fatty liver. According to the interview, some workers were former alcoholics, but the available health data shows that the person concerned has recovered. According to recommendations from health experts, if the workers are monitored and frequently consult a medical doctor, then the workers’ health condition should be under control.

Government employees who work at BATAN either as administrative staff, researchers, technicians, or managers of nuclear facilities follow the rules for paid civil servants. Therefore, the HRP participants follow the Indonesian Government's payroll system. In addition to their basic salary, they receive additional benefits called nuclear hazard benefits, functional benefits, and child benefits for those who have children. For the HRP participants, each employee would receive a total income of US$ 6000–8000 per year (depending on the position and seniority). For Indonesia's economic conditions, this income is sufficient for a small family as the average per capita income of the Indonesian population in 2018 was US$ 3800 [38]. However, there was 5 personnel who require attention from this financial indicator. One of the participants was experiencing financial problems because they have to provide financial support for the education of their relatives (this is the culture in several ethnic groups in Indonesia). Some participants also have many children and are paying their mortgage. These situations caused them to work elsewhere outside office hours to supplement their income. Security experts and technical experts stated that because the participants are skilled and dedicated workers, management is expected to continue to employ them in their respective positions. However, management needs to monitor their condition lest this financial problem interferes with their performance.

A work environment that emphasizes the honesty of each personnel encourages them to solve problems and improve performance continually. Encouraging all personnel to express their opinions will minimize the risk to the security and safety of nuclear facilities. On the other hand, if personnel are afraid to voice their honest opinions, this situation will become the forerunner of the problem. When an organization values the opinions of its employees, they feel a sense of ownership and are thus motivated to give their best effort. This sense of ownership allows them to share organizational goals and targets easily with the management team and work towards achieving them. A work environment that encourages open discussion also allows for workers to identify missteps and easily report them to their supervisor for their improvement. Such an environment facilitates creative problem solving as everyone can express their ideas and desire to find a solution instead of pointing fingers and blaming. Moreover, in a work environment that supports communication, workers can easily contribute innovative ideas to improve the organization.

During the interview, all HRP candidates answered all questions openly, thus demonstrating their honest nature. There was no fear of raising a complaint about a situation in the office that they deemed necessary to improve and they were also open to conveying their personal conditions. This situation showed that mutual trust has grown within the research reactor facility such that every person could convey any difficulties they have encountered in the workplace. This allows problems to be easily identified and for solutions to be immediately sought.

The most important reason for following organizational rules and regulations is personal and co-worker safety. This is especially important when working with risky facilities and instruments such as in research reactors or other nuclear facilities. Failure to properly turn off equipment or use safety features could result in injury or death. Like other nuclear research organizations, BATAN has regulations to protect the safety and security of its workers and facilities, therefore that failure to comply with these policies may result in personnel being sanctioned or terminated immediately.

All research reactors in Indonesia have been operational for more than 30 years and the operation records showed that there has never been an accident or significant anomaly that has posed a risk to safety and security. This data suggests that all personnel working in the research reactor follows the rules and regulations set by BATAN and the regulatory body. The result of the interviews and data on HRP candidate personnel also reflects this statement as the compliance of all personnel to the existing rules and regulations was found to be very high.

Next, social interactions at work can positively or negatively impact the perceptions of happiness and health behaviors. Feelings of comfort can be enhanced by work interactions where trust, collaboration, and positivity are present. When individuals feel valued and respected during social interactions, it would improve their perceived well-being. Conversely, wellness and health behaviors are negatively impacted when social interactions at work lacked a sense of trust, collaboration, positivity, and respect. They are also hindered when employees feel that their interactions lacked justice and empathy.

The HRP participants have worked together for at least five years, some for more than 20 years. In general, their social interactions are good and well established. Nevertheless, there was one person with a yellow label. During the interview, the person tended to answer normatively, he did not respond quickly and tended to be silent. However, he has a good work performance, was responsible, and has high skills and expertise in his field. Interestingly, his national insight score was also low (yellow label). Based on our observations of his social media, he liked content containing hate against the government and liked comments from groups that the government prohibits. In an in-depth interview, he briefly conveyed his political views, which tended to be oppositional, and he considered the government to be unfair in terms of economic, social, and political matters. The interviewer believes that this resulted from the polarization of the 2014 and 2019 presidential elections. However, he appeared to have no motive to pose an internal threat to the facility where he worked. Nevertheless, a follow-up is required as this individual may require special handling by security experts and psychologists. In the meantime, management has warned him about his social media behavior and still monitors his attitude and performance.

The following indicator is emotional stability. This may be a significant personality factor, but evidence suggests that external factors encourage and prevent this behavior. For example, being emotionally unstable can also result in job dissatisfaction. These factors, such as having a heavy workload, will increase job dissatisfaction and ultimately lead to burnout, which is associated with negative emotions. External stressors (e.g., divorce, death or illness in the family, financial problems) can also affect personnel performance and emotional stability. If these stress factors are not addressed, they can lead to destructive behavior in the workplace.

There were 6 HRP candidates with yellow labels. As many as 5 of them also had relatively low scores on the loyalty indicator (they expressed some job dissatisfaction), and coincidentally these 5 individuals were also under 30 years of age. This may be due to stronger emotions at a younger age. Getting older is associated with more positive general emotional well-being and better emotional stability, despite the problems at hand becoming more complex [39]. From the interviews, these 6 participants could easily react to topics that they considered unfair and untrue. Personnel assistance programs can help relieve these external stressors by providing services such as psychological counseling. Unfortunately, currently, there is no program for this type of personnel assistance at BATAN.

The next indicator assessed was self-awareness. It is a powerful experience that can help individuals understand who they are and why they do what they do. It also helps each person to become more responsible for their behavior and make improvements in life that support their wants. Lastly, it also helps organizations reduce incident rates. Being self-aware is very crucial when it comes to working. It is easy to become complacent and unhappy when a person is unsatisfied with their job. When workers do not like their jobs or cannot maximize their talents, they may be less and less motivated to do well. This situation can lead to poor performance and create a safety risk for themselves and those around them because they do not fully understand how their behavior impacts others. Therefore, a lack of self-awareness in employees can be a problem for organizations, as it affects productivity, work safety, and security.

As previously mentioned, some employees were dissatisfied at work, as shown by the loyalty indicators. They complained about the need for rewards for meritorious workers and the opportunity to learn and practice to increase their knowledge and abilities. Similarly, there were notes on emotional stability. However, the interviewers and the HRP team concluded that all HRP candidates were highly self-aware of who they were and what they worked for. These results indicate that these HRP candidates understand the targets that must be achieved for their work in the research reactor.

## 5 The future of HRP

BATAN developed the HRP by taking into account the situation and conditions in Indonesia. This program has been tested in several important positions in a research reactor as an effort to prevent insider threats at the research reactor facility at BATAN. The results of this program's first implementation were quite good. Nevertheless, improvements could be made by adding a sufficient number of experts in security, safety, health, and psychology, as well as improving the qualitative and quantitative analysis.

This study's satisfactory results were due to the relatively complete background data of BATAN workers as well as the excellent cooperation and communication of the HRP participants. They were very open and did not hesitate to share their views with the HRP implementation team. In the future, this program should be continued periodically or annually or if the observations of management or surrounding colleagues indicate unusual behavior among existing employees. Future implementations of the HRP should also significantly expand the number of participants, with a high priority given to high-risk positions at nuclear facilities, such as IT officers and even research reactor directors.

In addition, the organization's management can implement the HRP program for prospective employees who have just graduated from college and will be placed in certain positions at nuclear facilities. For these prospective employees, the HRP may be modified to a more simplified form, with a focus on indicators of work motivation and national insight. This action is a form of early prevention of any possible insider threats. However, the methods used must still foster the critical and creative nature of the personnel and do not interfere with the expression of individual freedom guaranteed by law.

**Fig. 2:**
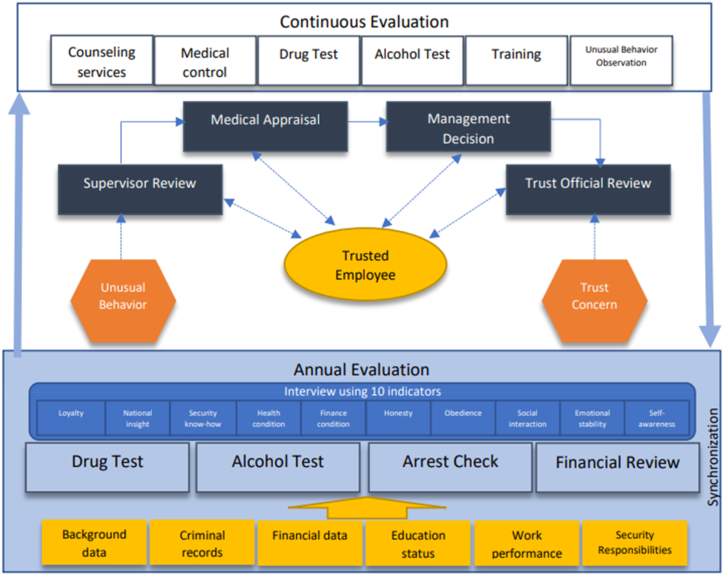
The Future HRP flowchart, modification from Ref. [16].

Fig. 2 depicts a concept of how to implement HRP at regular intervals and when there is an unusual behavior among personnel working at a nuclear facility. Given the vital position of religion for the Indonesian people [40], this concept can still be developed, for example, by inviting moderate religious experts to serve as consultants, especially in preventing the entry of radical or deviant thoughts that can affect the safety and security of workers, facilities, and the community.

On the other hand, the HRP implementation team must develop themselves to understand the behavior of personnel, including the use of social media, therefore it is necessary to involve a reliable psychologist. When needed, they can also act as a member of the counseling team to deal with any worker behavior problems.

Based on public surveys that have been conducted, the majority of Indonesian people support the operation of nuclear power plants (NPP) [41,42]. Meanwhile, the Government of Indonesia has made a roadmap toward the target of net zero emissions by 2060 and will operate the NPP in the 2040s so that this HRP program can serve as a model for recruitment, promotion, and review of employees in high-risk positions in the power plants.

## 6 Conclusion

BATAN attempted to develop and implement an HRP to determine the reliability of the personnel operating research reactors. It is BATAN's effort in implementing government regulations on safety and security at nuclear installations, nuclear energy regulatory agency regulations and physical protection conventions. The scope of the HRP was to analyze the workers' work reliability, measure their loyalty to institutions, and determine the national insight of personnel who are working in crucial positions in a research reactor in Indonesia. In addition to ensuring the safety and security of nuclear facilities from insider threats, this program is also essential for counteracting the radicalism that has recently emerged in Indonesian society.

All personnel who participated as HRP participants scored well and were not likely to be an insider threat. Nevertheless, the results indicate significant records of job dissatisfaction and require management's attention. The analysis showed that the organizational system must revise the business process regarding the Human Resources Department (HRD), such as by providing opportunities for employees to improve their knowledge and skills and providing rewards for those who excel.

Moreover, this study showed the need for a counseling room for employees. Counseling rooms are rarely available in government organizations in Indonesia, but for organizations that manage facilities that have safety and security risks, such as research reactors, it may be beneficial to prevent unwanted events.

Although it is unlikely that radicalism will affect the HRP participants, management still needs to continue to monitor some of the participants who were dissatisfied with government policies and tended to sympathize with banned groups. Management must warn them about the unwanted possibilities that may occur. If there is no other way, management must transfer that person to a lower-risk position.

The development of this HRP should be continued, especially by improving the HRP implementation team's quality and by expanding the scope of HRP participants to include a wider range of individuals that work in safety and security risk positions. In the future, the HRP can also be used as an assessment tool by the organization's human resource development program for promotions, rotations, and personnel transfers.

## Data availability statement

The data that has been used is confidential.

## Additional information

No additional information is available for this paper.

## Author contribution statement

All authors listed have significantly contributed to this article's development and writing. Djarot Sulistio Wisnubroto Conceived and designed the experiments; Analyzed and interpreted the data; Wrote the paper, Khairul Khairul Conceived and designed the experiments; Performed the experiments; Contributed materials, analysis tools, and data, Fatmuanis Basuki Conceived and designed the experiments; Performed the experiments; Contributed materials, analysis tools, and data, Endang Kristuti Analyzed and interpreted the data; Wrote the paper.

## Declaration of competing interest

The authors declare that they have no known competing financial interests or personal relationships that could have appeared to influence the work reported in this paper.
